# Biphonation in animal vocalizations: insights into communicative functions and production mechanisms

**DOI:** 10.1098/rstb.2024.0011

**Published:** 2025-04-03

**Authors:** Romain A. Lefèvre, Océane Amichaud, Doğa Özcan, Elodie F. Briefer

**Affiliations:** ^1^ Behavioural Ecology Group, Section for Ecology & Evolution, Department of Biology, University of Copenhagen, Copenhagen Ø 2100, Denmark; ^2^ INRAE, CNRS, Université de Tours, PRC, Nouzilly 37380, France; ^3^ Molecular Biology and Genetics Department, Faculty of Engineering and Natural Sciences, Bahcesehir University, Istanbul 34353, Turkey

**Keywords:** vocal communication, nonlinear phenomena, biphonic calls, vocal production, vertebrate

## Abstract

Biphonation, defined as the simultaneous production of two distinct, non-harmonically related fundamental frequencies, has traditionally been viewed as an anomaly or a by-product of vocal pathology. Recent studies have challenged this assumption and found that biphonic calls are present in the natural vocalizations of a wide range of taxa, including birds, amphibians and mammals. This phenomenon could play an essential role in communicating distinct pieces of information at short- versus long-distance, increase call complexity to allow more individually distinct calls, and provide cues to the sender’s direction of movement. Proposed mechanisms underlying biphonation production include asymmetries in vocal fold oscillations, the addition of aerodynamic whistles, the involvement of secondary structures, and bilateral specializations. This scoping review underscores the adaptive significance of biphonic calls in non-human animals, highlighting their role in the evolution of vocal communication and suggesting avenues for future research.

This article is part of the theme issue ‘Nonlinear phenomena in vertebrate vocalizations: mechanisms and communicative functions’.

## 1. Introduction

Our understanding of animals' vocalizations is heavily rooted in the source–filter theory, which suggests that sound production involves a bipartite mechanism: the source and the filter. The source (the larynx in mammals) produces sound through vocal fold vibrations as air is projected from the lungs. Then, this produced sound undergoes modulation by the filter system—including the vocal tract and oral and nasal cavities—that shapes the acoustic signal by selectively amplifying or attenuating frequencies [1]. This theoretical framework has been instrumental in delineating the relationship between the anatomical features of vocal apparatuses and the acoustic characteristics of the emitted sounds, shedding light on the evolutionary trajectories and functional aspects of vocal communication [2,3].

**Figure 1.**
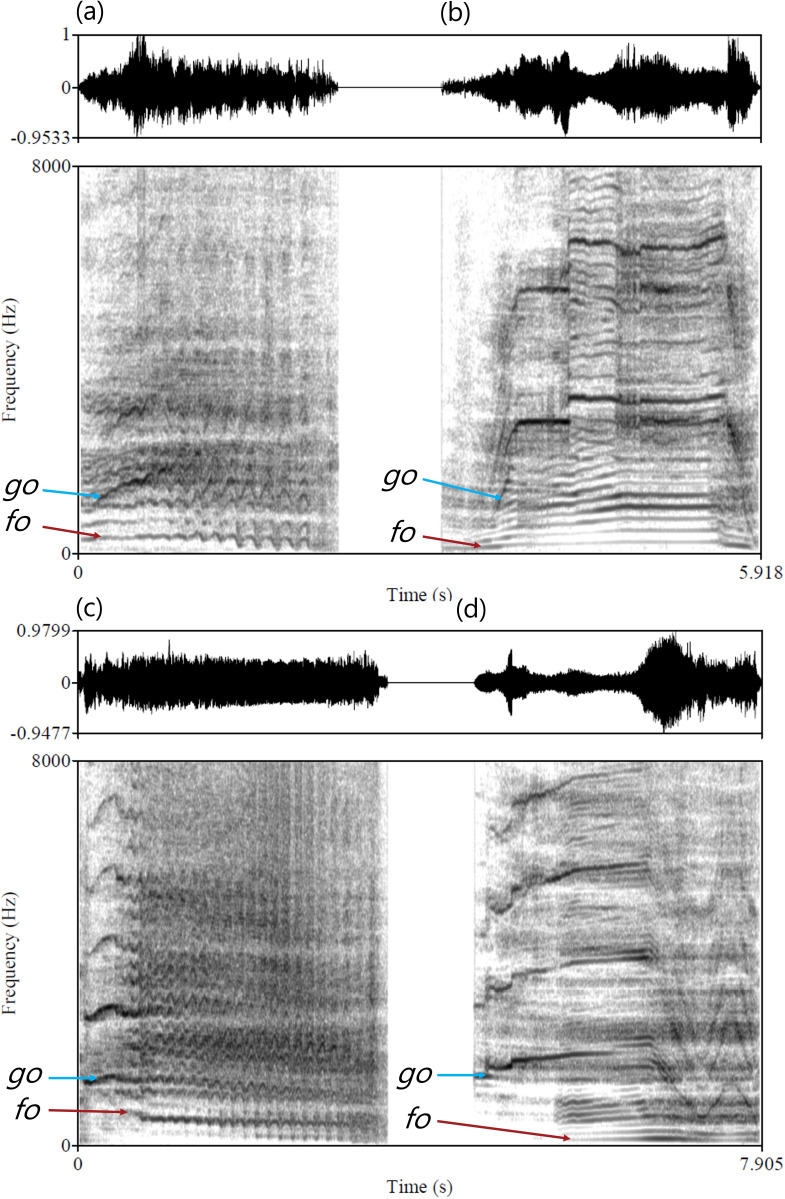
Biphonation in horse and wapiti vocalizations. Spectrograms (below) and oscillograms (above) of two horse whinnies (*a*) and (*c*), and two wapiti bugles (*b*) and (*d*), showing biphonation. The lowest of the two fundamental frequencies (*f*_o_) and the highest one (*g*_o_) are indicated with red and blue arrows, respectively. (*a*,*b*) Examples of calls where *f_o_* and *g_o_* fully overlap in time; (*c*,*d*) examples of calls where *g_o_* starts before *f_o_* (wapiti bugles were provided by Megan Wyman, University of Zurich).

More recently, studies have also revealed that vocal production apparatuses can produce complex, often unpredictable patterns that arise from the interactions between its different components. These patterns are classified as ‘nonlinear phenomena’ (NLPs), a term that includes a variety of distinct acoustic features such as subharmonics (i.e. additional frequency components occurring below the fundamental frequency), deterministic chaos (i.e. nonrandom noise linked to highly irregular vibrations of oscillators) and biphonation, a ‘quasi-periodic’ phenomenon [4,5]. The term biphonation is derived from Greek, where *bi* signifies *two* and *phone* means *voice* or *sound*. Characterized by the concurrent production of two—or even sometimes three—distinct, overlapping fundamental frequencies (hereafter ‘*f*_o_*’* and *‘g*_o_*’*) within a single vocalization, biphonation comprises a lower frequency component, often corresponding to the fundamental frequency of vocal fold vibration and a higher frequency component that is harmonically unrelated (figure 1) [4,6]. Initially perceived as a mere by-product lacking direct adaptive significance, early observations of biphonation were predominantly associated with vocal pathologies in both humans (e.g. infants and adults manifesting vocal disorders [6,7]) and various animal species, including Japanese macaques (*Macaca fuscata* [8]), domestic dogs (*Canis familiaris* [9,10]), and domestic cats (*Felis catus* [11]). However, more recent studies challenge this perspective [12], highlighting the occurrence of biphonic calls in the vocal repertoires of numerous species, including marine mammals [13–15] and terrestrial mammals [16–19], but also many birds [20,21] and amphibians [22]. Whether it arises from the control of different vibratory modes of a single anatomical source within a bird’s vocal organ, the syrinx [21], or from two distinct sources involving a potential whistle-like mechanism [18], biphonation reflects an interesting capability in the evolutionary biology of animal communication.

The aim of this scoping review is to provide a broad, although not systematic, overview of biphonation in animal vocalizations. Specifically, our goal is to synthesize key studies on non-human animals, in order to understand (1) the potential communicative functions of biphonic calls and (2) their possible production mechanisms. By reporting insights from studies in taxa exhibiting this NLP, we aimed to underscore the adaptive significance of biphonation and demonstrate its role in enriching animal communication systems, as well as provide an overview of the important trends, gaps and future research directions of this field. Finally, we aim to reach a consensus on the definition of this phenomenon that could be applied across species and provide clear guidelines for its use.

## 2. Communicative functions

Despite its widespread occurrence across the animal kingdom, studies specifically investigating the possible communicative functions and adaptive significance of biphonation (i.e. aside from other NLPs are rare. While several possible functions have been proposed in the literature, this review focuses on the three most prominent ones, which we detail in the following sections (table 1).

**Table 1.** Functions proposed for biphonation, along with the species in which each function has been proposed, percentage of calls showing biphonation and corresponding references.

function	species	call type and percentage	reference(s)
communicating multiple pieces of information	North American wapiti (*Cervus canadensis*)	100% bugles [18]	[18]
	domestic horse (*Equus ferus caballus*)	100% whinnies [19]	[19]
enhancing individual or group identity	dhole (*Cuon alpinus*)	20–92% of yap–squeak sequences [16]	[23]
	emperor penguin (*Aptenodytes forsteri*)	100% display calls [24]	[20]
	bottelnose dolphins (*Tursiops truncatus*)	1 of 42 signature whistles [15]	[15,25]
	short-finned pilot whales (*Globicephala macrorhynchus*)	0–57% of the calls [26]	[26]
	killer whales (*Orcinus orca*)	89% of vocalizations [27]	[14,28]
	three-spined toadfish (*Batrachomoeus trispinosus*)	4/723 hoots and 12/519 grunts [29]	[29]
providing cues to caller’s direction of movement	killer whales (*Orcinus orca*)	89% of vocalizations [27]	[30]
	dhole (*Cuon alpinus*)	20–92% of yap–squeak sequences [16]	[31]

### (a) Communicating multiple pieces of information

Across species, an inverse correlation exists between body size and the fundamental frequency of vocalizations, with large African elephants (*Loxodonta africana*) known to produce calls with an *f_o_* as low as 16.8 Hz on one side of the spectrum [32] and small rainforest bats (*Kerivoula pellucida*) whose buzz calls can attain a maximum frequency of 250 kHz on the other side [33]. However, this assumption often does not hold when comparing individuals of the same species, sex and age, which can be attributed to the fact that surrounding bones do not constrain laryngeal structure growth. This allows variation in *f_o_* that is not correlated with body size [34], but mainly with vocal fold tension and length [35], or that is generated through other mechanisms such as resonances and air sac modulation. Notably, two species deviate from this acoustic allometry by producing much higher frequencies than predicted by their body size: domestic horses (*Equus ferus caballus*) and North American wapiti (*Cervus canadensis*) (see Fig. 8.3 in [3]). Indeed, despite their considerable body mass, these two species were initially thought to produce unusually high fundamental frequencies [36,37]. However, further investigations have shown that their high fundamental frequency is accompanied by a lower fundamental frequency within the range predicted by acoustic allometry [18,19] (see figure 1 for spectrograms illustrating these two frequencies). In both species, the potential function of this biphonation has been suggested to be the simultaneous communication of multiple messages [18,19].

Horse whinnies typically start with a high fundamental frequency (*g_o_*) (mean ± s.d. = 1543.26 ± 326.45 Hz, range: 493−3012 Hz), to which a lower fundamental frequency (*f_o_*) is added later on (399.22 ± 99.39 Hz, range: 52−1050 Hz; figure 1*a*,*c*) [19]. The ratio between these two frequencies differs between horses, and their contours are only partially correlated within whinnies (significantly in 63 of 71 analysed whinnies, with *r*^2^ = 0.51 ± 0.23, range = 0.06–0.92 [19]), suggesting real biphonation. Acoustic analyses of whinnies produced in emotionally positive contexts (reunion with one or several group mates) compared with negative contexts (separation from one or several group mates) showed that these two partially independent fundamental frequency components provide insight into the animal’s emotional state [19]. Interestingly, each frequency provides a distinct message: *f_o_* reflects one of the two main emotional dimensions, the animal’s emotional arousal (bodily activation assessed based on the horse’s heart rate). At the same time, *g_o_* indicates the second main emotional dimension, emotional valence (negative/unpleasant in separation versus positive/pleasant in reunion). This communication of separate emotional dimensions in the two frequencies could play a crucial role in regulating social dynamics and facilitating interactions between horses [19].

Male wapitis produce sexual calls known as bugles, characterized by the simultaneous production of a low *f_o_* in the range of 76 to 250 Hz and a higher ‘whistle’ component, *g_o_*, ranging between 145 and 4187 Hz (figure 1*b*,*d*) [18]. These two frequencies are not harmonically related, can vary in opposite directions, and sometimes also appear separately, suggesting real biphonation and distinct production mechanisms [18]. The low *f_o_*, which is characterized by a dense spectrum of harmonics highlighting formant frequencies, has been proposed to convey information about body size to surrounding listeners, playing a significant role in mating and dominance displays, but limited to close range owing to its relatively low amplitude. Concurrently, the high-frequency *g_o_* component is optimized for long-distance transmission owing to its much higher amplitude, announcing the presence of male wapitis across more considerable distances. The presence of biphonation in this species could thus constitute an adaptive strategy in reproductive contexts to concurrently broadcast size-related cues at close distances through *f_o_* and signal presence over long distances through the high-amplitude *g_o_* component [18].

### (b) Enhancing individual and group recognition

The second function proposed for biphonation, which has been suggested for several species (table 1), is to facilitate individual or group recognition by increasing the number of individuals or groups that can be distinguished through more complex signals. Dholes (*Cuon alpinus*) produce 11 types of calls, one of which has a biphonic structure: the yap–squeak [16]. This call type simultaneously merges a high-frequency squeak at around 5 kHz (*g_o_*), and a low-frequency yap at around 1 kHz (*f_o_*), and is produced along with non-biphonic squeaks and yaps during peaceful interactions with group members. Volodina *et al.* [23] explored the potential function of these biphonic calls through detailed acoustic analyses. Comparison of the variation occurring between individuals in squeaks, yaps and biphonic yap–squeaks revealed that yap–squeaks are much more individualized (96.7% correct assignment in a discriminant function analysis) compared with squeaks (80.7%) and particularly yaps (44.7%) [23]. The integration of two frequencies in yap–squeaks was thus hypothesized to enhance individual recognition, especially within the constraints of dholes’ densely vegetated habitats and large social groups. These peaceful short-distance calls could thus function to maintain stable social relationships within packs [23]. While this hypothesis is compelling, playback experiments would be necessary to validate whether the increased individuality in these calls has practical implications for communication and social interactions in this species.

Recent studies have also documented NLPs in the vocalizations of female concave-eared torrent frogs (*Odorrana tormota*), which were found to be as complex as those of males, with numerous calls exhibiting complex upward/downward frequency modulations, and 39% of female calls containing at least one component of NLPs, including biphonation [22]. These nonlinear characteristics and individual signatures suggest that female frogs might use such vocalizations for individual recognition, similar to their male counterparts [38], during sexual advertisement and mate attraction.

In birds, the presence of a ‘two-voice phenomenon’ is common as their vocal organ, the syrinx, contains one to three sound sources depending on the species, allowing the simultaneous production of two independent fundamental frequencies [39]. The display call of emperor penguins (*Aptenodytes forsteri*) is composed of a series of syllables characterized by the presence of two interacting fundamental frequencies and their respective stack of harmonics [24]. In this species, the lack of visual cues and the necessity of leaving eggs and chicks in dense groups of adults make auditory signals the primary means of individual identification and location. The function of biphonation in emperor penguins has been investigated through detailed acoustic analysis, playback experiments using natural and modified calls, and propagation of synthetic sounds [20]. This study revealed that the two frequencies are necessary for individual recognition. The authors propose that the presence and interaction between the two frequencies create a vast number of possible vocal signatures necessary for discriminating individuals in large colonies. In addition, these two fundamental frequencies, and especially the lower one, propagate well, facilitating the transmission of the sound within the colony. Therefore, the biphonic structure enriches the calls with unique acoustic signatures that facilitate individual recognition of partners, mates, parents and offspring. This is vital for maintaining social bonds and coordinating reproductive behaviours in densely populated colonies [20]. A similar biophonic structure can be found in the display calls of king penguins (*Aptenodytes patagonicus*) [40], suggesting that biphonation could be a characteristic of the genus *Aptenodytes,* which does not nest and lives within large colonies in Antarctica [41].

Bottlenose dolphins’ (*Tursiops* sp.) signature whistles—individually distinctive and stereotyped signals used for individual recognition [42,43]—can sometimes be biphonic, although this seems to be a rare phenomenon. For instance, among 42 signature whistles identified by Papale *et al.* [15] in Sicily, only one showed two simultaneously produced and independent fundamental frequencies. Based on the production context, the authors suggested that this particular whistle, encountered 13 times, represented an individual and not a group recognition signal. Similarly, only one of the 28 signature whistles identified by Kriesell *et al.* [25] in Walvis Bay was biphonic. A function of biphonation in enhancing individuality has also been suggested for short-finned pilot whales (*Globicephala macrorhynchus*), in which two-component calls were encountered in 0–57% of the calls produced by tagged animals [26]. Biphonation in odontocetes, although not present in all vocalizations, could thus be used by some individuals to enhance their distinctiveness.

Biphonation has also been suggested to enhance group recognition in killer whales (*Orcinus orca*) [14,28]. In this species, both monophonic (containing one fundamental frequency, range: 80−2400 Hz) and biphonic calls that contain an overlapping, much higher, fundamental frequency (range: 2−12 kHz) are produced during intragroup communication [30,44]. Monomorphic calls were shown to have a lower mean apparent source level, suggesting a function for closer-range communication, compared with biphonic calls, which could function for longer-distance communication [45]. Filatova *et al.* [28] investigated the possible function of these biphonic calls further by comparing their context of production with the number of pods present and their activity. The authors found an increased prevalence of biphonic calls in mixed-pod associations, while monomorphic calls were mainly used during intra-pod communication. This suggests a possible role of biphonic calls for affirming pod and matriline affiliations and enhancing group recognition in contexts with higher risks of confusion [28]. This hypothesis was later confirmed by Filatova [14], who showed that the presence of both frequency components improves call classification to the correct family.

Finally, three-spined toadfish (*Batrachomoeus trispinosus*) have been reported to produce vocalizations with nonlinear acoustic complexity similar to that found in tetrapods. In toadfish, the bilaterally separated swim bladder enables the production of biphonation, defined as a ‘two-voice’ system similar to the one found in songbirds. In toadfish, biphonic calls are believed to enhance the spectro-temporal content and complexity of the vocal signals, facilitating individual recognition, and as a strategy to differentiate agonistic from advertisement calls produced during territorial displays [29].

### (c) Providing cues to caller’s direction of movement

In addition to enhancing group recognition, biphonation in killer whale calls has been suggested to provide listeners with information about the caller’s direction [30]. Indeed, Miller [30] showed that variation in the energy distribution between the high- and low-frequency components of biphonic calls changed when the killer whales moved in the direction of the hydrophone compared with away from it. This difference in propagation occurs because high-frequency sounds are more directional and tend to lose energy faster in the water owing to higher absorption rates. In contrast, low-frequency sounds propagate more uniformly and can travel longer distances with less attenuation [46]. This difference of more than 10 dB in relative energy between the lower and higher frequency components in killer whale calls could thus provide information about the direction of movement of the caller with respect to the receiver and hence facilitate coordination and spacing regulation between individuals [30].

A similar function was suggested in dholes, where the ratio of the sum of amplitudes higher than 5 kHz to the sum of amplitudes lower than 5 kHz was observed to be higher when animals were running towards compared with away from the microphone [31]. Dholes’ diphonic yap–squeaks could thus provide enhanced information about movement directionality [31]. However, similar results were found for yaps, which also contain a rich spectrum of frequencies but are not biphonic. Since yaps do not contain any information about individuality [16], the authors suggest that the presence of two frequencies in yap–squeaks could simultaneously transmit multiple information, with the high-frequency component aiding in identifying the caller and the low-frequency element providing crucial orientation information. This segregation of information, aligning with the multiple information hypothesis (§2a), could ensure the maintenance of pack dynamics and cohesion, even in environments where direct visual contact is hindered [23].

## 3. Mechanisms of production

Although production mechanisms underlying biphonation have only been demonstrated in a few species, several mechanisms have been suggested based on anatomical and acoustic analyses. Below, we describe the four primary mechanisms that are supported by a substantial body of literature supports.

### (a) Irregular spatio-temporal vibration patterns of vocal folds

One of the earliest proposed mechanisms for biphonation in humans is left–right asymmetry in vocal fold oscillations, where the left and right vocal folds vibrate differently, producing two independent fundamental frequencies [6,47]. This desynchronization between vibratory modes of the vocal folds can occur naturally, or be pathological, such as in left recurrent nerve paralysis [48]. Alternatively, owing to stiffness imbalance between the anterior and posterior sides of the vocal folds, desynchronized anterior–posterior vibratory modes can lead to anterior–posterior biphonation [47]. When the stiffness asymmetry reaches a certain level, the drop in spatial coherence desynchronizes the vibration modes and results in biphonation [49]. Such asymmetries tend to produce *f_o_* and *g_o_* frequencies that are close to each other [7].

### (b) Vortex shedding and aerodynamic whistles

The second mechanism proposed for biphonation is through the creation of vortices by the constriction of the airstream at narrowings present in the upper airways, such as occurs during human whistling, which could generate a second, higher fundamental frequency (*g_o_*). In a study on anaesthetized domestic dogs, whines were presumed to be produced through a vortex-shedding mechanism with circular airflow patterns; as air from the lungs is forced through the vocal folds, vortices are formed, and a high fundamental frequency is produced [50]. Such a mechanism can also occur further up, at the level of narrowings in the nasal tract, as proposed to produce the *g_o_* component in dhole’s biphonic calls [51]. In North American wapiti, anatomical investigations and acoustical modelling suggested that the high-frequency *g_o_* component could be a vortex-induced (i.e. aerodynamic) whistle produced at the level of the nostrils [18]. Finally, vortex-induced vibrations have been suggested as one possible mechanism underlying the production of biphonation or even triphonation in human contemporary vocal music [52].

### (c) Involvement of secondary structures

A third mechanism giving rise to biphonic vocalizations could be the presence of anatomical specializations in the larynx or vocal tract that could sustain periodic oscillations and hence produce a second, higher fundamental frequency (*g_o_*). Sykes' monkeys (*Cercopithecus mitis albogularis*) produce unusually high-frequency tonal screams, with no evidence of acoustic energy below 6−8 kHz [53]. To produce vocalizations with such high frequencies, animals of that size would have either to reduce their vocal tract length from around 6.5 to 1 cm, which is a physiologically impossible manipulation based on the anatomical constraints of their vocal apparatus, or to produce a very high fundamental frequency equal to the lowest band in the signal [53]. However, both scenarios likely require anatomical adaptations that would be atypical of primate vocal anatomy. Acoustic and laryngeal measurements suggest that the vocal membranes (vocal fold extensions) may vibrate independently from the vocal folds to produce sounds with unusually high fundamental frequencies [53]. Alternatively, high fundamental frequencies may be generated through the controlled alternation of oscillations of the vocal membranes and the primary vocal fold tissue, as involved in the production of squeals [53]. In such a system, with two sources vibrating out of phase, the fundamental frequency is higher than the oscillation rate of any component of the larynx [53]. More recently, the high-frequency squeak of Asian elephants was reported to be produced by lip buzzing; as air is forced through the tensed lips, a self-sustained lip vibration produces a high frequency [54]. Other sounds, such as snorts, can be produced simultaneously through other sources (e.g. trunk), leading to biphonation.

### (d) Bilateral specializations

The last primary mechanism proposed for biphonation is the involvement of bilateral specializations. In toothed whales, independently controlled phonic lip pairs located in the nose enable these animals to use different vocal registers on each side, analogous to human vocal fry and falsetto, and resulting in the production of both low-frequency signals and high-frequency echolocation clicks [55]. Notably, the distinction between right and left phonic lips, speculated to produce different sound types, could give rise to the biphonic calls observed, for example, in killer whales or dolphins [25,28].

As mentioned in the previous sections, a similar ‘two-voice phenomenon’ is commonly observed in birds. Unlike most animals that produce sounds using structures in the mouth or throat, birds generate vocalizations in the syrinx, a specialized song organ located at the junction where the trachea divides into the two primary bronchi [39]. The syrinx of songbirds, for instance, contains two sets of individually controlled ‘vocal folds’, named the medial and lateral labia, which produce sound using the same physical mechanism observed in mammals (‘myoelastic–aerodynamical theory’) [56,57]. This arrangement provides them with the ability to produce two non-harmonically related frequencies simultaneously [21].

## 4. Discussion

Our review consolidates current knowledge on biphonation, highlighting its occurrence not as a mere vocal anomaly but as a significant phenomenon within the acoustic repertoires of various species. Although the function of biphonation has been rarely addressed or tested, it may allow animals to communicate multiple messages simultaneously through relatively independent frequencies (*f_o_* and *g_o_*), which can be used for short- versus long-range communication depending on their relative frequency and amplitude [18,19]. Second, the presence of two fundamental frequencies and their corresponding harmonics could increase signal complexity, enhancing individual or group signatures [20,23]. Last, biphonic calls, owing to their wide frequency range, could provide cues to the sender’s direction of movement through the relative amplitude ratios between *f_o_* and *g_o_* as the sender approaches or moves away [30,31]. These various functions are not mutually exclusive, and multiple factors might explain the evolution of biphonation in different species. Various production mechanisms have been proposed, including asymmetrical vocal fold oscillations, aerodynamic whistles and secondary structures (e.g. vocal membranes or bilateral specializations). Below, we provide advice for identifying biphonation, and ruling out alternative explanations, and suggest a definition applicable across species.

### (a) Identifying biphonation on spectrograms

To identify biphonation—the simultaneous presence of two fundamental frequencies (*f_o_* and *g_o_*) that are not integer multiples of each other (e.g. 1/2 or 1/3 [4,6])—on a spectrogram, several criteria can be considered: (1) *f_o_* and *g_o_* should have different contours and vary independently, forming non-parallel spectral bands that sometimes go in opposite directions and cross each other (figure 1*a*,*b*); (2) the ratio of *f_o_* to *g_o_* should thus not be an integer (which could indicate that *f_o_* is, in fact, a subharmonic of *g_o_*) and vary within and between vocalizations; (3) *f_o_* and *g_o_* should overlap in time at some point during the vocalization. However, instances where they can be observed separately (figure 1*c*,*d*) provide further evidence that they vary independently and suggest that they result from distinct production mechanisms.

### (b) Distinguishing biphonation from other nonlinear phenomena

It is essential to distinguish biphonation from other NLPs, as several other NLPs might resemble biphonation on a spectrogram. First, one can rule out a register transition—an abrupt change in fundamental frequency [58]—by verifying that *f_o_* and *g_o_* do overlap in time at some point in the vocalization. Second, as mentioned above, the ratio and correlation between *f_o_* and *g_o_* should be investigated to ensure that they are not harmonically related and that *f_o_* is not a subharmonic of *g_o_* [12]. Third, amplitude modulation (AM) or frequency modulation (FM), corresponding to cyclic changes of the waveform amplitude envelope (for AM) or of a fundamental frequency (for FM), can produce sidebands departing at equal distance from each side of the fundamental frequency and its harmonics on a spectrogram, when the modulation frequency (e.g. *f_o_*) is a lot lower than the other frequency (e.g. *g*_o_) [4,5]. This phenomenon often occurs together with biphonation, in cases where *f_o_* modulates *g_o_* [4,18]. For that reason, the presence of sidebands has, in some studies, been used as a cue to detect biphonation (e.g. [27]). However, since sidebands can also occur when there is only one fundamental frequency (and AM and/or FM), we suggest attempting to distinguish between these two NLPs when possible: (1) ‘real biphonation’, which explicitly involves the simultaneous production of two distinct, non-harmonically related fundamental frequencies, and can be accompanied by sidebands or not; and (2) sidebands due to AM or FM, in the absence of two non-harmonically related fundamental frequencies. For instance, their trace on the spectrogram can sometimes be quite distinct: sidebands tend to appear as two bands of lower amplitude, situated at equal distances above and below a fundamental frequency and each of its harmonics, while biphonation appears as non-parallel spectral bands that can vary independently [4,6]. Sound modifications, such as the removal of the AM, can also be used to verify that biphonation is present [19].

In general, choosing the right spectrogram settings can help to distinguish biphonation from other NLPs [59]. Depending on the settings used, cases of biphonation can also be missed. This can, for example, occur if broadband spectrograms are used with a high-frequency *g_o_* and very low-frequency *f_o_* (below 50−100 Hz), leading to the appearance of *f_o_* as a pulse-train structure (with pulses corresponding to the vibrations of the oscillators), which might resemble chaos. By contrast, the same *f_o_* will appear as more linear with visible harmonics in a narrow-band spectrogram (e.g. see figure 1 in [60] or descriptions of the low-frequency component of killer whale vocalizations in [13]).

### (c) Distinguishing biphonation from artefacts

In addition to the NLPs mentioned above that can be confounded with biphonation, spectrogram artefacts should also be ruled out. These include aliasing, which arises when the recording sampling frequency is set too low and frequency components of the vocalization occur above one-half of it. As a result, image frequencies of these high components appear on the spectrogram and can resemble biphonation [4]. Lastly, reverberations caused by resonance properties of the environment produce artificial sound prolongation, which, if they overlap with the following vocalization, can be confused with biphonation (see [19] for an example of how to rule out alternative explanations).

### (d) The ‘two-voice phenomenon’, a special case of biphonation?

In the case of songbirds, a distinction has been made between biphonation involving the involuntary production of two independent frequencies through the same sound source (i.e. one side of the syrinx in birds) and the ‘two-voice phenomenon’ consisting of the controlled production of two frequencies by distinct sound sources (i.e. each side of the syrinx) [21]. However, these two phenomena are complex to distinguish, and excluding ‘controlled’ cases necessitates a deep understanding of the vocal production mechanisms of studied species, which is hard to achieve for wild animals. For instance, Zollinger *et al.* [21] could do so only after carefully monitoring respiratory pressure and airflow on each side of the syrinx. In addition, these terms have been used interchangeably in other species (e.g. in marine mammals in some studies owing to uncertainties about precise production mechanisms [15,27,28]; but see [13]), and this distinction has, to our knowledge, not been applied in the human voice literature. We therefore suggest considering all cases where two—or even sometimes three—independent fundamental frequencies overlap in time as biphonation, regardless of whether the underlying mechanisms are distinct and whether the production is voluntary or due to irregularities.

One concern with the two-voice phenomenon is that, besides its ‘controlled’ aspect mentioned above, two independent fundamental frequencies and their respective stack of harmonics—hence two periodic sounds—are present in the signal. This raises the question of whether these cases should still be classified among ‘NLPs’ (e.g. [27]). However, even with two separate sources, interactions between these frequencies, such as modulation of one by the other, often occur (wapiti [18]; killer whales [27]). For these reasons, biphonation is considered a ‘quasi-periodic’ phenomenon [61]. Therefore, we propose that all cases of simultaneous production of two independent fundamental frequencies should be considered as an NLP.

### (e) Anatomical interactions

A common criterion for biphonation is that the two fundamental frequencies should be independent. However, as mentioned above, complete independence is rare. When the two frequencies are produced at the same source, such as in the case of left–right asymmetry in vocal fold oscillations, interactions between the two folds still occur [62]. Similarly, when two different sources produce the two frequencies, these are usually located within the vocal apparatus and, hence, anatomically close to each other. Many interaction effects can thus occur, such as sidebands at linear combinations of *f_o_* and *g_o_*, arising owing to the amplitude modulation of *g_o_* by *f_o_* [18]. Since these two frequencies can modulate each other, we suggest that partial independence of *f_o_* and *g_o_* is enough to be considered biphonation.

### (f) Towards a consensus on the definition of biphonation in animal vocalizations

Given the considerations mentioned above, we propose that biphonation should be defined as the production, by single or separate sources, of two (or more) partially independent, not harmonically related, fundamental frequencies overlapping in time over at least part of the vocalization. This definition includes instances where one frequency appears to modulate the other. This approach ensures clarity and consistency in classifying vocal phenomena across species. This definition embraces the complexity of biphonic calls across species, thus stimulating research across the animal kingdom. Future research should explore the occurrence and functions of biphonation across more species to elucidate the evolutionary pathways and ecological pressures that shape this particular NLP. Finally, we encourage the use of controlled playback experiments with synthetic vocalizations to help decipher the communicative significance of biphonic calls.

## Data Availability

This article has no additional data.
